# Effect of adding a mobile health intervention to a multimodal antimicrobial stewardship programme across three teaching hospitals: an interrupted time series study

**DOI:** 10.1093/jac/dkx040

**Published:** 2017-02-28

**Authors:** E. Charani, M. Gharbi, L. S. P. Moore, E. Castro-Sanchéz, W. Lawson, M. Gilchrist, A. H. Holmes

**Affiliations:** 1 NIHR Health Protection Research Unit in Healthcare Associated Infection and Antimicrobial Resistance at Imperial College London, Du Cane Road, London W12 0NN, UK; 2 Imperial College Healthcare National Health Service Trust, St Mary’s Hospital, Praed Street, London W2 1NY, UK

## Abstract

**Objectives:** To evaluate the impact of adding a mobile health (mHealth) decision support system for antibiotic prescribing to an established antimicrobial stewardship programme (ASP).

**Methods:** In August 2011, the antimicrobial prescribing policy was converted into a mobile application (app). A segmented regression analysis of interrupted time series was used to assess the impact of the app on prescribing indicators, using data (2008–14) from a biannual point prevalence survey of medical and surgical wards. There were six data points pre-implementation and six data points post-implementation.

**Results:** There was an increase in compliance with policy (e.g. compliance with empirical therapy or expert advice) in the two specialties of medicine (6.48%, 95% CI = −1.25 to 14.20) and surgery (6.63%, 95% CI = 0.15–13.10) in the implementation period, with a significant sudden change in level in surgery (*P *<* *0.05). There was an increase, though not significant, in medicine (15.20%, 95% CI = −17.81 to 48.22) and surgery (35.97%, 95% CI = −3.72 to 75.66) in the percentage of prescriptions that had a stop/review date documented. The documentation of indication decreased in both medicine (−16.25%, 95% CI = −42.52 to 10.01) and surgery (−14.62%, 95% CI = −42.88 to 13.63).

**Conclusions:** Introducing the app into an existing ASP had a significant impact on the compliance with policy in surgery, and a positive, but not significant, effect on documentation of stop/review date in both specialties. The negative effect on the third indicator may reflect a high level of compliance pre-intervention, due to existing ASP efforts. The broader value of providing an antimicrobial policy on a digital platform, e.g. the reach and access to the policy, should be measured using indicators more sensitive to mHealth interventions.

## Introduction

Technology in healthcare is constantly evolving. The application of innovation and information technology has proliferated in every aspect of healthcare delivery from using robotics to deliver non-invasive surgical interventions, to electronic medical records, and prescribing systems.^1–3^ With the advent of smartphones, and their ubiquitous use by healthcare professionals, a new technological platform has been introduced into healthcare. Increasingly, mobile health (mHealth) is being used to deliver an array of different interventions through smartphone applications (apps) targeting both healthcare professionals and healthcare users.^4^^,^^5^ In healthcare, the development and implementation process of medical device technologies is regulated, monitored or researched; however, for information systems, e.g. electronic prescribing and smartphone technology, there has to date been little regulation or governance.^6^ It is only in the last couple of years that the need for governance on the use of smartphone apps in healthcare has been recognized.^7^

In the field of antimicrobial prescribing, where the drive to address suboptimal antimicrobial prescribing in secondary care has led to the development of many interventions, the use of clinical prescribing tools and decision support systems has been well documented.^8–11^ Such systems have been added to existing antimicrobial stewardship programme (ASP) interventions in diverse ways, ranging from pharmacy purchasing and ordering systems, to electronic prescribing systems with specific add-ons targeting antimicrobial prescribing, to providing access to susceptibility data.^12^ Although they have shown some impact on tackling inappropriate antimicrobial prescribing in hospitals, data on the long-term sustainable impact of such systems or their effect on patient outcomes is lacking.^8^^,^^13^ It was to this plethora of electronic interventions in healthcare, and in particular, in antimicrobial prescribing, that mHealth technology was added. In the last 10 years the number of healthcare-related smartphone apps has grown exponentially. In 2014 it was estimated that there were over 40 000 medical- and health-related apps available.^14^ In previous studies we have reported on the frequent use of smartphone apps by healthcare professionals during clinical consultations.^15^^,^^16^ In this paper we report the effect of introducing a smartphone decision support tool for antimicrobial prescribing on prescribing trends in secondary care. This study builds on existing work we have conducted in this field on the development of clinical decision support systems to support antimicrobial prescribing.

## Methods

### Setting

This study was performed across the three main hospitals of Imperial College Healthcare Trust (ICHT) Hospitals in west London. Across the sites there are 1300 beds. At the time of the study the hospitals did not have any electronic prescribing systems.

### Ethics

Approval to conduct this study was obtained, via e-mail, by the ICHT and Imperial College London joint research office (approval reference not applicable). All data used were anonymized, and all data were stored and analysed in secure computers within the organization.

### Intervention

**Figure 1:**
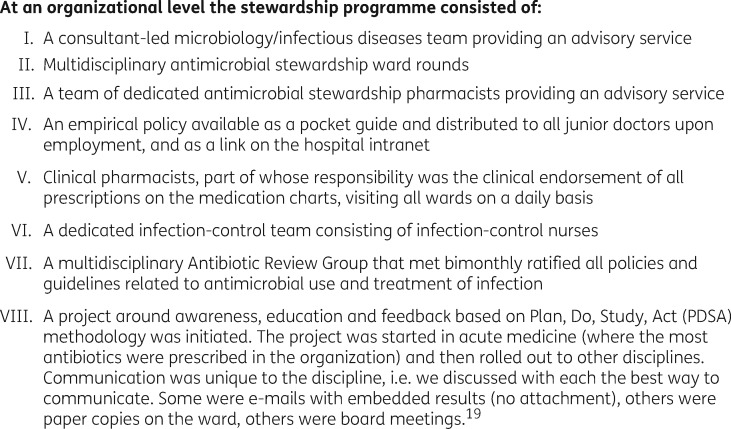
Description of all the antimicrobial stewardship interventions in place during the time period of this study.

The study extends over a 6 year period, 3 years pre-intervention and 3 years post-intervention roll-out in August 2011. The hospitals already had a multimodal ASP in place (Figure 1). In addition to this, the hospitals are affiliated with an academic research unit dedicated to research in antimicrobial stewardship. The Imperial antibiotic prescribing policy application (IAPP) was developed as part of a collaboration and partnership between the academic and clinical staff across Imperial College. The IAPP was launched across the hospitals in August 2011. The purpose of developing the app was to make the antimicrobial prescribing policy available at the point of care. The IAPP was developed following a baseline study with multi-professional stakeholder engagement investigating the prevalence of the usage of smartphones amongst healthcare professionals.^15^^,^^16^ A multimodal dissemination strategy was used in the introduction and implementation of the IAPP, with four communication channels: (i) teaching; (ii) e-mails; (iii) the intranet homepage; and (iv) the ICHT newsletter.^15^ The IAPP replaced a pocket guide that was no longer produced or disseminated.

Post-IAPP, the ASP already described continued. In 2012 the UK Department of Health published the ‘Start Smart Then Focus’ guidance for improving the quality of antimicrobial prescribing in hospitals.^17^ The key elements of this guidance, which included steps for reviewing patients who were prescribed therapeutic doses of antibiotics, were incorporated in the updated version of the IAPP as pop-up prompts, launched in June 2012. Between its launch in 2011 up to 2014, the IAPP underwent several iterations, each time adding functionality in response to user feedback.

### Point prevalence data

Data from the biannual point prevalence survey (PPS) that is conducted across the hospitals were used for the interrupted time series (ITS) analysis in this study. Clinical pharmacists use a standardized, protocol-driven data collection form to collect the data from patient medication charts on wards. All patients in hospital on the day of data collection are included in the study. Data are collected from all patients receiving systemic antimicrobials. The key prescribing indicators the PPS measures are: (i) adherence of choice of antimicrobial to local policy or microbiology/infectious disease team recommendation; (ii) documentation of indication on medication charts; and (iii) documentation of stop or review date on the medication charts. These three indicators (choice, indication and stop or review date for prescribed antimicrobials) were chosen as indicators to observe trends in prescribing pre- and post-IAPP adoption. The impact of using mobile apps on antibiotic prescribing outcomes has not been previously reported. In the absence of direct outcome measures that can be linked to the effect of the intervention, we used these existing, routinely collected data on antibiotic prescribing outcomes as ‘proxy indicators’, or representative outcomes measures of the impact of the IAPP.

Data from general medical and surgical patients were analysed separately because at the time the IAPP was launched the ASP was more focused on medicine. Within medicine, data from haematology, renal medicine and oncology were excluded from the analysis as these specialties were recognized to have specialist policies separate from the empirical general policy for the treatment of infections, and benefited from more targeted specialist input from infectious disease and microbiology services. Data from ICUs were also excluded from the analysis as antimicrobial therapy for patients in intensive care is under daily review by infectious disease and medical microbiology staff.

### Data analysis

A segmented regression analysis of ITS was used to evaluate the impact of the IAPP. Six-monthly PPS data for the selected indicators from October 2008 to June 2014 were plotted. The clearly defined intervention period (August 2011) and the availability of at least three data points before, and three data points after the intervention enabled this quasi-experimental design.^18^ The analysis estimated the intervention effect whilst taking account of time trend and autocorrelation among the observations. The ITS allowed the estimation of any sudden change in level immediately after the intervention, which is defined as the difference between the observed level at the first intervention time point and that predicted by the pre-intervention time trend, the estimation of the difference between pre- and post-intervention slopes, and the estimation of the level effect 6, 12 and 24 months post-intervention. The level effect at 6, 12 and 24 months post-implementation is the difference between the predicted value 6, 12 and 24 months post-intervention calculated with the pre-slope and the observed value 6, 12 and 24 months post-implementation.^8^^,^^19^ After testing the absence of first-order autocorrelations with the Durbin–Watson statistic, a time series regression model, ARIMA (autoregressive integrated moving average), without adjustment for autocorrelation was fitted to the PPS data. STATA version 12 (STATA Corp, College Station, TX, USA), statistical software was used to perform the analysis.

## Results

**Figure 2:**
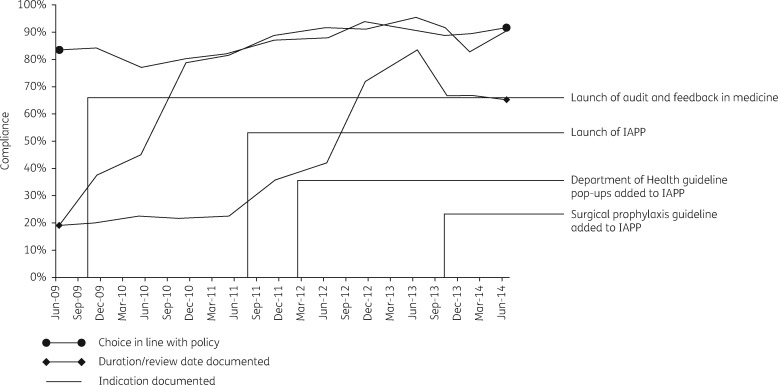
Timeline of PPS data for the three indicators; the launch of the Department of Health’s ‘Start Smart Then Focus’ guidelines and the addition of the surgical prophylaxis policy to the IAPP are indicated.

**Figure 3:**
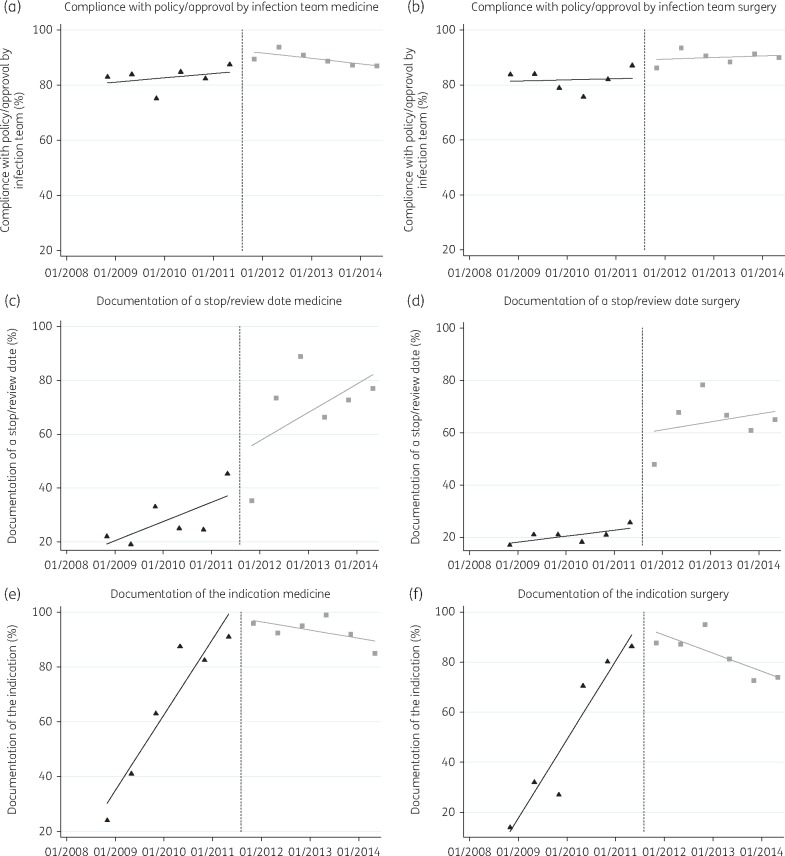
Observed trends plotted as percentage change (*y*-axis) over time (*x*-axis) in the point prevalence indicators pre- and post-IAPP launch for medicine and surgery specialties. Triangles = before intervention. Squares = after intervention. Broken line = intervention. Continuous lines = fitted values.

The timeline of the various interventions together with the launch of the IAPP is plotted in Figure 2 along with the trends in the three indicators measured in the biannual PPS (aggregated data from medical and surgical specialties). There was a positive, upwards trend for all the indicators prior to the launch of the IAPP (Figure 3).

In the pre-implementation period, compliance with the policy for the antibiotics prescribed was already high across both medicine and surgery (Table 1 and Figure 3a and b). In the implementation period of the IAPP there was an increase in percentage compliance with policy in both medicine (6.48%, 95% CI = –1.25 to 14.20) and surgery (6.63%, 95% CI = 0.15–13.10), the change in level was significant in surgery, but not in medicine (Table 1). The level effect remained positive in both medicine and surgery at 6 and 12 months.

**Table 1:** A summary of the ITS analysis on the percentage change in the three measured indicators from the PPS in medical and surgical specialties

		Pre-implementation slope, % (95% CI)	Implementation period sudden change in level, % (95% CI)	Change in slope pre- and post- implementation, % (95% CI)	6 month level effect, % (95% CI)	12 month level effect, % (95% CI)	24 month level effect, % (95% CI)
Medicine	compliance with policy/approval by infection team	0.79 (−0.78 to 2.36)	6.48 (−1.25 to 14.20)	−1.77 (−6.36–2.82)	4.70 (−3.55–12.96)	2.93 (−7.96 to 13.83)	−0.61 (−19.21 to 18.00)
	documentation of stop/review date	3.57 (−6.99 to 14.12)	15.20 (−17.81 to 48.22)	1.69 (−13.27 to 16.66)	16.90 (−23.39 to 57.18)	21.22 (−46.54 to 88.98)	21.98 (−54.88 to 98.84)
	documentation of indication	13.83 (10.89–16.77)**	−16.25 (−42.52 to 10.01)	−15.32 (−22.88 to − 7.77)**	−31.58 (−53.55 to − 9.60)**	−46.90 (−66.65 to − 27.14)**	−77.55 (−100.94 to − 54.15)**
Surgery	compliance with policy/approval by infection team	0.21 (−1.29 to 1.71)	6.63 (0.15 to 13.10)*	0.084 (−3.15 to 3.31)	6.71 (−0.06 to 13.48)	6.80 (−1.60–15.20)	6.96 (−6.55–20.48)
	documentation of stop/review date	1.15 (−10.62 to 12.92)	35.97 (−3.72 to 75.66)	0.36 (−11.94 to 12.67)	36.33 (−13.96 to 86.63)	36.70 (−24.84 to 98.24)	37.43 (−47.49 to 122.34)
	documentation of indication	15.71 (8.16–23.25)**	−14.62 (−42.88 to 13.63)	−19.31 (−30.38 to − 8.24)**	−33.93 (−65.37 to − 2.50)*	−53.24 (−90.97 to − 15.52)**	−91.86 (−146.90 to − 36.83)**

* *P *<* *0.05. **P <* *0.01.

Documentation of a stop/review date had a lower baseline level in the pre-intervention period (Figure 3c and d). In the implementation period, there was an increase in level in both medicine (15.20%, 95% CI = −17.81 to 48.22) and surgery (35.97%, 95% CI = −3.72 to 75.66) (Table 1). The change in slope pre- and post-intervention as well as the level effect at 6, 12 and 24 months post-IAPP were positive, but not significant.

Documentation of indication had significantly increased in both medicine and surgery in the pre-implementation period. In the post-implementation period, both slopes in medicine and surgery changed significantly, resulting in a decrease in the documentation of the indication with a negative sudden change in level of −16.25% (95% CI = −42.52 to 10.01) in medicine; and −14.62% (95% CI = −42.88 to 13.63) in surgery. The change in percentage of prescriptions with an indication remained negative but significant at 6, 12 and 24 months post-implementation in both medicine and surgery.

## Discussion

The introduction of the IAPP had a positive effect on compliance with policy indicators in both medicine and surgery, as shown by the sudden change in levels. In surgery the improvement was statistically significant. The reasons for the observed effect in surgery may be that most ASP interventions prior to the IAPP had focused on medicine. As a result, this new intervention and decision support system may have had a greater effect in the surgical teams as there were fewer other interventions that may have saturated the effect. The baseline compliance with the empirical policy was high in the study and this may be due to the existing and ongoing high-level ASP activities across the organization. The IAPP improved (shown by the sudden change in level) the documentation of the stop/review date in both medical and surgical specialties, but this improvement was not significant. The documentation of indication had improved throughout the study, independent of the IAPP intervention, and the introduction of the IAPP had a negative (but not significant) effect, as shown by the sudden change in level. One reason for this may be that, since early 2010, clinical pharmacists across the study hospital network were required to endorse medication charts, and as part of the clinical responsibility to ensure judicious antimicrobial prescribing would document the indication onto the drug chart if the prescriber has not done so.

Using the PPS data as an indicator for antimicrobial prescribing trends pre- and post-IAPP was possible because of the consistency and the reliability of the PPS data. It also enabled the mapping of other ASP interventions that were taking place across the organization. The three indicators have been collected consistently since 2008.

Identifying and isolating the appropriate outcome measure for mHealth interventions in the context of an evolving and multifaceted ASP is challenging. There is growing interest in the adoption of mHealth in ASPs. Adoption of such interventions is an inevitable progression of technology in healthcare. Any evaluation should be done in the context of the added value that they will have to existing ASPs. Perhaps in organizations where the ASP is being newly developed, the addition of smartphone apps may add significant value. The cost-effectiveness of such interventions versus other well-established components of ASP also requires further evidence and evaluation. Staff may be more amenable to using mHealth to gain access to booklets or pocket guides on local policies. The WHO has recently published guidelines for reporting health interventions using mobile phones.^20^ One of its key recommendations when developing mHealth interventions is the need to clarify what the added value of such interventions is to existing systems. The IAPP was introduced in 2011 at a time when using mHealth in healthcare was still a relatively new concept. The aim was to make the local empirical antimicrobial prescribing policy more accessible to staff and with the help of decision algorithms to encourage optimized antimicrobial prescribing. Data on the roll-out and initial use of IAPP have been published and the subsequent qualitative impact of the app reported.^15^ However, developing a study that measures the added effect of a clinical decision support system to an existing multifaceted antimicrobial stewardship model across a multi-site healthcare organization is more challenging. This may be the case for any system where there is a saturation of interventions targeting a single outcome, e.g. ASP. In the context of this study electronic prescribing was not implemented at the time of the implementation of the IAPP. It was precisely because of lack of a uniform electronic prescribing system that the idea of a decision support tool delivered through smartphone apps was considered to be a possible way to provide point-of-care access to the existing empirical policy.

The indicators used in this study were part of a larger and more complex ASP in a real-life clinical setting. As such, establishing causality between the implementation of the IAPP and the observed trends in prescribing is not possible. The indicators chosen did not measure the wide-ranging impact of the IAPP. The mechanisms to measure and describe the utility of such mHealth interventions should be multimodal, and sensitive enough to measure the added value in the context of multiple interventions aimed at maximizing outcome measures. We would advocate integrating quantitative measures such as those presented here with qualitative criteria such as increased access to the policy at the point of care, the reach of the policy to healthcare professionals and the feedback from users.^15^^,^^21^ In this study, the addition of the antimicrobial prescribing policy as an mHealth app to a multifaceted ASP did not demonstrate a significant change in antimicrobial prescribing trends. The added value of the IAPP has been in the reach and access to the antimicrobial prescribing policy amongst a wider range of staff across our organization.^15^ Healthcare organizations aiming to implement apps as part of ASPs should consider the evidence presented here in order to manage any expectations they might have of the impact of apps on antimicrobial prescribing trends.
